# Longitudinal assessment of interstitial lung abnormalities on CT in patients with COPD using artificial intelligence-based segmentation: a prospective observational study

**DOI:** 10.1186/s12890-024-03002-z

**Published:** 2024-04-23

**Authors:** Yusuke Shiraishi, Naoya Tanabe, Ryo Sakamoto, Tomoki Maetani, Shizuo Kaji, Hiroshi Shima, Satoru Terada, Kunihiko Terada, Kohei Ikezoe, Kiminobu Tanizawa, Tsuyoshi Oguma, Tomohiro Handa, Susumu Sato, Shigeo Muro, Toyohiro Hirai

**Affiliations:** 1 Department of Respiratory Medicine, Graduate School of Medicine, Kyoto University, Kyoto, Japan; 2 Department of Diagnostic Imaging and Nuclear Medicine, Graduate School of Medicine, Kyoto University, Kyoto, Japan; 3 Institute of Mathematics for Industry, Kyusyu University, Fukuoka, Japan; 4 Respiratory Medicine and General Practice, Terada Clinic, Himeji, Hyogo Japan; 5 Department of Respiratory Medicine, Kyoto City Hospital, Kyoto, Japan; 6 Department of Advanced Medicine for Respiratory Failure, Graduate School of Medicine, Kyoto University, Kyoto, Japan; 7 Department of Respiratory Care and Sleep Control Medicine, Graduate School of Medicine, Kyoto University, Kyoto, Japan; 8 Department of Respiratory Medicine, Nara Medical University, Kashihara, Nara Japan; 9 Department of Respiratory Medicine, Graduate School of Medicine, Kyoto University, 54 Kawahara-cho, Shogoin, Sakyo-ku, 606-8507 Kyoto, Kyoto, Japan

**Keywords:** Artificial intelligence, CT, COPD, Interstitial lung abnormality

## Abstract

**Background:**

Interstitial lung abnormalities (ILAs) on CT may affect the clinical outcomes in patients with chronic obstructive pulmonary disease (COPD), but their quantification remains unestablished. This study examined whether artificial intelligence (AI)-based segmentation could be applied to identify ILAs using two COPD cohorts.

**Methods:**

ILAs were diagnosed visually based on the Fleischner Society definition. Using an AI-based method, ground-glass opacities, reticulations, and honeycombing were segmented, and their volumes were summed to obtain the percentage ratio of interstitial lung disease-associated volume to total lung volume (ILDvol%). The optimal ILDvol% threshold for ILA detection was determined in cross-sectional data of the discovery and validation cohorts. The 5-year longitudinal changes in ILDvol% were calculated in discovery cohort patients who underwent baseline and follow-up CT scans.

**Results:**

ILAs were found in 32 (14%) and 15 (10%) patients with COPD in the discovery (*n* = 234) and validation (*n* = 153) cohorts, respectively. ILDvol% was higher in patients with ILAs than in those without ILA in both cohorts. The optimal ILDvol% threshold in the discovery cohort was 1.203%, and good sensitivity and specificity (93.3% and 76.3%) were confirmed in the validation cohort. 124 patients took follow-up CT scan during 5 ± 1 years. 8 out of 124 patients (7%) developed ILAs. In a multivariable model, an increase in ILDvol% was associated with ILA development after adjusting for age, sex, BMI, and smoking exposure.

**Conclusion:**

AI-based CT quantification of ILDvol% may be a reproducible method for identifying and monitoring ILAs in patients with COPD.

**Supplementary Information:**

The online version contains supplementary material available at 10.1186/s12890-024-03002-z.

## Background

Smoking and aging cause a variety of chronic respiratory diseases, such as chronic obstructive pulmonary disease (COPD) and interstitial lung disease (ILD). COPD is pathologically characterized by alveolar destruction, known as emphysema, and small airway disease, leading to persistent airflow limitations and respiratory symptoms [1], while ILD is characterized by inflammation and fibrotic changes in the lung parenchyma [2]. Although the underlying pathophysiology of ILD is different from that of COPD, the coexistence of emphysema and ILD is common and known as combined pulmonary fibrosis and emphysema (CPFE) [3, 4]. This condition is associated with a high incidence of lung cancer and pulmonary hypertension, leading to a poor prognosis [3, 5], but its pathogenesis remains unknown.

In contrast with histological analyses in which coexistence of emphysema and fibrosis is easily observed [6], fibrotic changes in emphysematous regions are subtle and cannot be resolved on high-resolution computed tomography (HRCT). However, interstitial lung abnormalities (ILAs) can be incidentally identified on CT in a subgroup of patients with COPD [7]. The presence of ILAs in patients with COPD has a negative impact on clinical outcomes [8, 9], and indeed, it is possible that ILAs are a precursor of CPFE. Therefore, the early detection of ILAs and assessment of their progression, particularly using an objective and reproducible quantification method, are crucial.

As the need for computer-aided CT analysis for ILD has increased, various methods of quantitative CT analysis have emerged, such as densitometry, texture analysis, and artificial intelligence (AI)-based methods [10]. AI-based image analysis has advantages in terms of high reliability and reproducibility over other methods. Furthermore, indices derived from AI-based image analysis have been associated with pulmonary function decline, exacerbation, and prognosis in ILD [11–13]. Artificial intelligence based quantitative CT image analysis software (AIQCT) is an AI-based image analysis software that can automatically classify chest CT images into normal lung, ground-glass opacities (GGOs), reticulations, consolidations, honeycombing, nodules, hyperlucencies, interlobular septum, bronchi, and vessels [14]. Both normal lung regions and the bronchus volume quantified using AIQCT have been associated with a poor prognosis in patients with idiopathic pulmonary fibrosis (IPF) [14]. However, the utility and applicability of AIQCT for quantifying ILAs in patients with COPD remains unclear.

Therefore, this study aimed to test the utility of AIQCT in evaluating the presence of ILAs in two independent COPD patient cohorts as well as ILA development using the longitudinal data from one of the cohorts. Furthermore, the study explored abnormal CT patterns associated with the emergence of ILAs.

## Methods

### Study population and longitudinal analysis

**Fig. 1:**
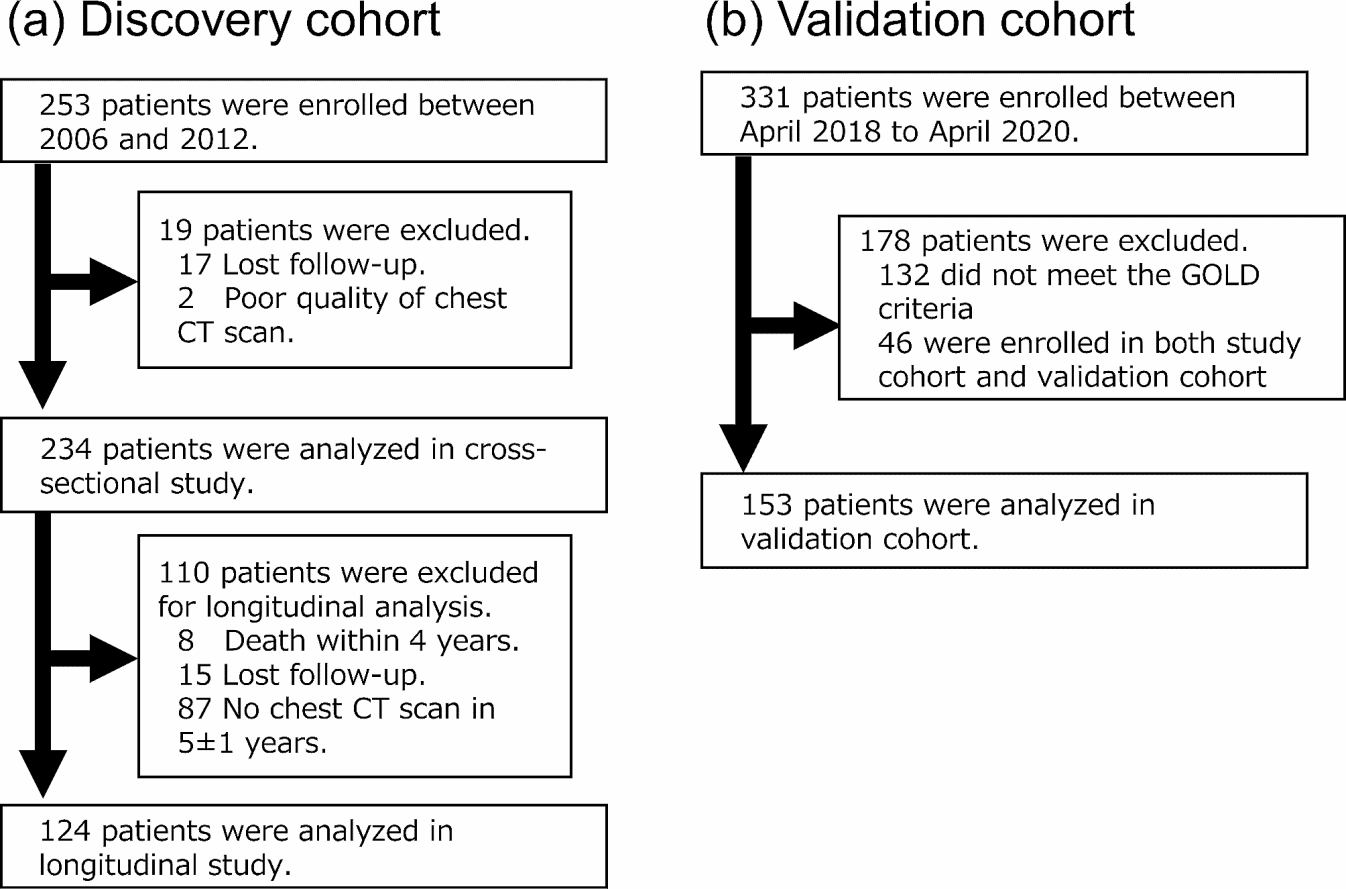
Patient flow chart Legend: (Fig. 1. **a**) Discovery cohort, (Fig. 1. **b**) Validation cohort

This is a retrospective analysis of two prospective observational cohorts of patients with COPD [15–18]. The discovery cohort included subjects who were older than 40 years, had a smoking exposure of more than 10 pack-years and were diagnosed with COPD according to the GOLD guidelines (forced expiratory volume in 1 s (FEV_1_)/forced vital capacity (FVC) less than 0.7 with respiratory symptoms) at Kyoto university hospital in 2006 to 2012. The exclusion criteria were (1) a diagnosis of ILD, (2) a prior history of lobectomy, and (3) inadequate CT images for quantitative analysis due to abnormal shadows such as pleural effusion, pneumonia, or poor quality. In addition to cross-sectional analysis, longitudinal analysis was performed on those who underwent chest CT scans within 5 ± 1 years from baseline (shown in Fig. 1). The validation of the thresholds of AIQCT indices for detecting ILA was performed using an independent dataset from another cohort [18]. The validation cohort was a prospective observational cohort from Kyoto university hospital and a respiratory clinic (Terada Clinic), consisting of smokers aged ≥ 40 years with a history of ≥ 10 pack-years between April 2018 and April 2020. The details of the validation cohort are described in the additional file 1.

### CT acquisition and visual assessment of ILAs

In the discovery cohort, all chest CT scans were acquired at full inspiration using an Aquilion 64 (sharp kernels, 0.5 mm slice thickness, 120 kVp, and autoexposure control; Cannon Medical Systems, Otawara, Japan). In the validation cohort, all chest CT scans were also acquired using an Aquilion Presicion scanner or an Aquilion lightning scanner (sharp kernels, 1.0 mm slice thickness, 120 kVp, and autoexposure control; Cannon Medical Systems, Otawara, Japan). Among one chest radiologist with 16 years’ experience and two CT-experienced pulmonologists with ≥ 8 years’ experience, two visually assessed ILAs in chest CT images according to the Fleischner Society statement [7]. Any cases of discordance were settled by a chest radiologist or consultation between the two raters. The patients’ clinical information was blinded, and the baseline and follow-up images were not compared when assessing the follow-up CT scans.

### Quantitative CT analysis using AIQCT

Quantitative analysis of the chest CT scans was performed using AI-based image analysis software named AIQCT, which was originally developed in collaboration with FUJIFILM Corporation to quantify parenchymal and airway abnormalities in IPF [14]. AIQCT uses a network architecture based on U-Net and automatically classifies structures on chest CT images into the following groups: normal lung, ground-glass opacities (GGOs), reticulations, consolidations, honeycombing, nodules, hyperlucencies, interlobular septum, bronchi, and vessels. According to a previous report [12], interstitial lung disease volume (ILDvol) was defined as the combined volume of honeycombing, reticulations, and GGOs. The absolute ILDvol and the percentage of ILDvol to total lung volume (ILDvol%) were analyzed in this study.

### Statistical analysis

Data are presented as the mean ± standard deviation (SD) unless otherwise indicated. The kappa coefficient was calculated to assess interrater reliability in the visual assessment of ILA [19]. Receiver operating characteristic (ROC) curve analyses and the Youden index were used to obtain the optimal threshold of ILDvol% in each lung volume fraction for detecting ILA. The areas under the ROC curves (AUCs) were compared using the DeLong test, and p values were corrected by the Bonferroni method. To test whether the diagnostic performance could be improved, sensitivity analysis was done in which the lung volume was equally divided into three or ten parts along the craniocaudal axis, and the highest ILDvol in each fraction was used in ROC curve analysis. Longitudinal changes in AIQCT indices were compared between subjects with new ILAs and those consistently without ILAs using the t test. Furthermore, adjusted mean differences in AIQCT indices were calculated, including age, sex, BMI, smoking status, and cumulative smoking exposure as covariates. Statistical analyses were performed using R statistical software version 4.3.1.

## Results

### Study population and quantified CT features

**Fig. 2:**
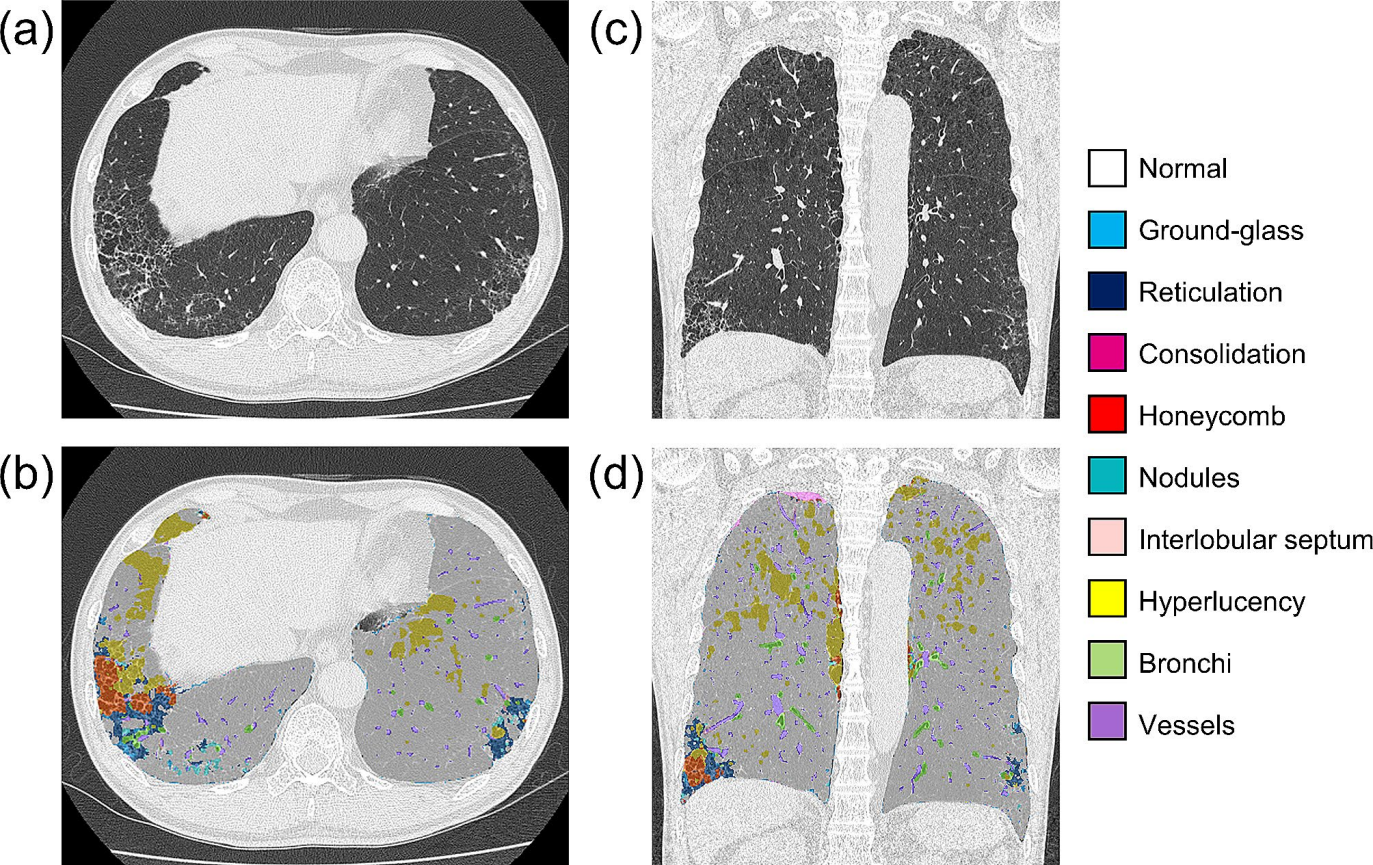
CT images of COPD with ILAs and corresponding AIQCT images Legend: The axial CT image of a 68-year-old male patient with COPD and ILAs in the discovery cohort (Fig. 2. **a**) and the corresponding AIQCT image overlaid on the original image (Fig. 2. **b**). Sagittal image of the same patient (Fig. 2. **c**) and the corresponding AIQCT image (Fig. 2. **d**). The ILD volume was 150 mL (2.90%)

Among 234 subjects, 32 (14%) had ILAs in the discovery cohort, and 15 out of 153 patients (9.8%) had ILAs in the validation cohort (Table 1). Interrater reliability for the visual assessment of ILAs was fair to moderate (kappa coefficient = 0.323–0.525, agreement = 81–88%). Compared to subjects without ILAs, those with ILAs were older and showed milder airflow limitation and a deteriorated diffusion capacity (Table 2). Table 2 also shows the AI-based quantification of CT features. The subjects with ILAs had higher incidences of honeycombing, reticulations, and GGOs than those without ILAs. Furthermore, nodule and bronchi volumes were greater in those with ILAs than those without ILAs. The clinical and radiological characteristics of the validation cohort are described in Table S1. Representative images of the AIQCT analysis are shown in Fig. 2.

**Table 1:** Patient characteristics in the two cohorts

	Discovery cohort		Validation cohort	
n	234		153	
Age, y	69.9	± 8.2	71.9	± 8.3
Sex, male	215	(91.9%)	142	(92.8%)
BMI, kg/m^2^	22.0	± 2.9	23.1	± 3.8
Pack-years	65.3	± 34.4	58.9	± 30.0
Smoking status, past	177	(75.6%)	111	(72.5%)
mMRC, ≥2	58	(24.8%)	37	(24.2%)
FVC, L	3.22	± 0.76	3.04	± 0.92
% predicted	93.2	± 16.7	87.4	± 21.9
FEV_1_, L	1.62	± 0.62	1.66	± 0.68
% predicted	60.4	± 19.6	62.4	± 23.1
FEV_1_/FVC, %	49.8	± 13.0	54.0	±12.0
Visual finding of ILAs	32	(13.7%)	15	(9.8%)

Data are the mean ± standard deviation or the number of patients with percentage in parentheses. *BMI* body mass index; *mMRC>* modified British Medical Research Council Dyspnea scale; *FVC* forced vital capacity; *FEV*_1_, forced expiratory volume in 1 s; *ILA* interstitial lung abnormality

**Table 2:** Clinical and radiological comparisons between patients with and without ILA in the discovery cohort

	No ILA		ILA		P value
n	202		32		
Age, y	69.4	± 8.1	73.1	± 8.0	0.016
Sex, male	185	(91.6)	30	(93.8)	0.945
BMI, kg/m^2^	21.9	± 2.8	22.7	± 3.4	0.155
Pack-years	64.5	± 34.7	70.8	± 32.0	0.338
Smoking status, past	150	(74.3)	27	(84.4)	0.309
mMRC, ≥2	47	(23.3)	11	(34.4)	0.258
FVC, L	3.22	± 0.77	3.18	± 0.72	0.77
% predicted	93.0	± 16.8	94.5	± 16.0	0.63
FEV_1_, L	1.59	± 0.62	1.80	± 0.56	0.065
% predicted	59.0	± 19.5	69.6	± 17.6	0.004
FEV_1_/FVC, %	48. 8	± 13.0	56.3	± 10.7	0.002
D_LCO_, mL/min/mmHg	13.2	± 5.2	11.1	± 4.5	0.03
% predicted	60.1	± 21.8	52.1	± 20.1	0.054
Normal lungs, mL	4175	± 997	3488	± 876	< 0.001
ILD volume, mL	51.1	± 37.2	114.1	± 76.6	< 0.001
Ground-glass opacities, mL	43	± 32	66	± 66	0.001
Reticulations, mL	7.0	± 8. 5	37.6	± 30.0	< 0.001
Consolidations, mL	7.7	± 3.9	8.8	± 3.4	0.141
Honeycombing, mL	1.0	± 1.7	10.0	± 18.9	< 0.001
Nodules, mL	5.7	± 4.9	11.7	± 9.6	< 0.001
Hyperlucencies, mL	821	± 1046	870	± 1058	0.806
Bronchi, mL	76.7	± 19.8	89.7	± 17.3	0.001
Vessels, mL	210	± 54	198	± 57	0.246

Data are the mean ± standard deviation or the number of patients with the percentage in parentheses. *ILA* interstitial lung abnormality; *BMI* body mass index; *mMRC* modified British Medical Research Council Dyspnea scale; *FVC* forced vital capacity; *FEV*_1_, forced expiratory volume in 1 s; *DLCO* diffusing capacity of the lung for carbon monoxide; *ILD* interstitial lung disease

### Diagnostic performance of AIQCT indices

By using ROC curve analysis, the optimal threshold of ILDvol% on whole lung for identifying ILAs was determined. As shown in Figure S1, the optimal threshold of ILDvol% on whole lung was 1.203%, and the area under the ROC curve was 0.863, with 87.5% sensitivity and 77.2% specificity. In sensitivity analysis using ILDvol% within a third or tenth of the whole lung volume, the sensitivity and specificity were also high, but the diagnostic performance was not improved (shown in Fig. S1). When the optimal threshold of ILDvol% on whole lung was applied to the validation cohort, the diagnostic performance was good (sensitivity was 99.3% and specificity was 76.3%).

### Longitudinal changes in the visual assessment of ILAs and AIQCT indices

**Fig. 3:**
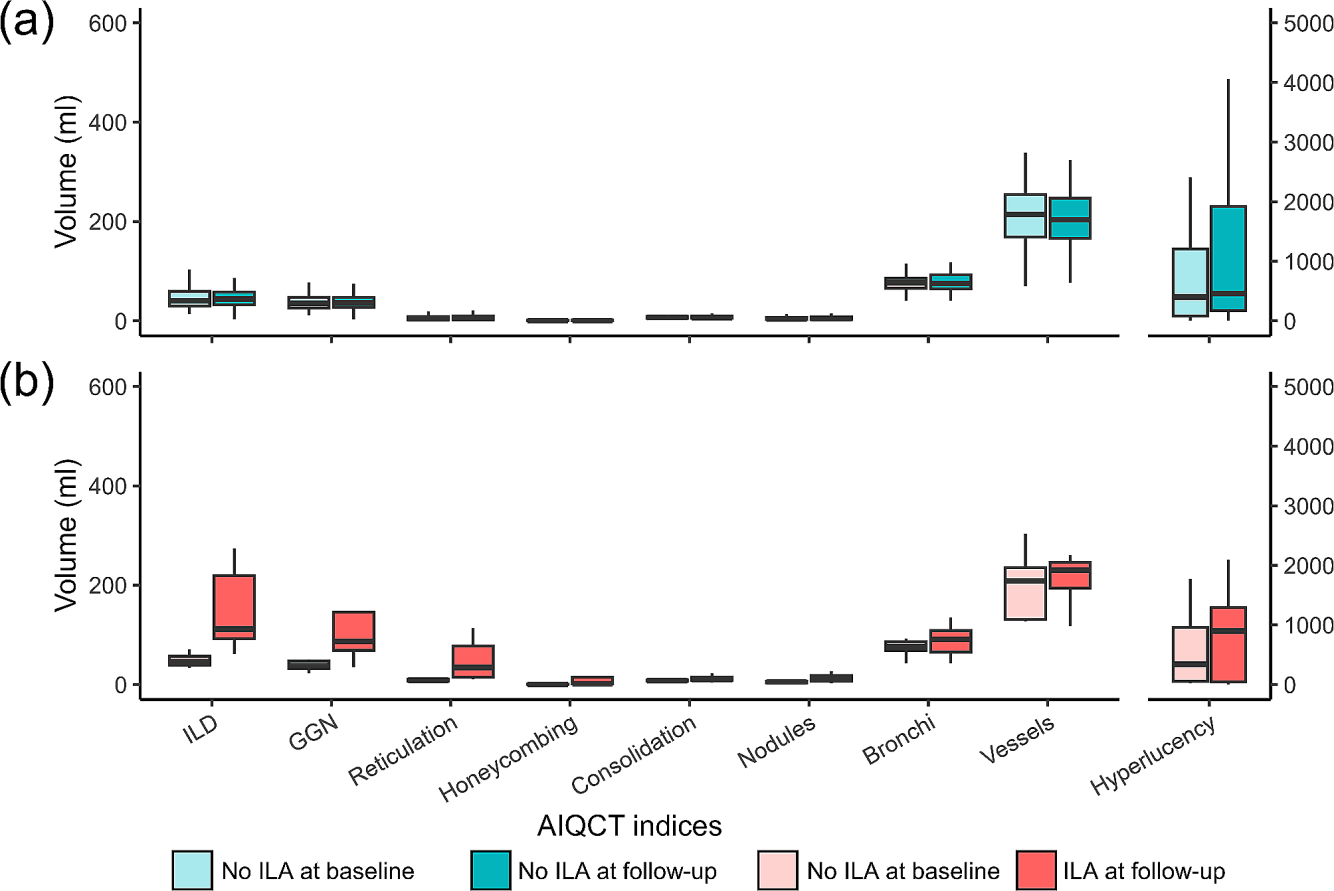
Longitudinal changes in AIQCT indices in patients consistently without ILAs and those with new ILAs. Legend: Each AIQCT index is shown at baseline and at follow-up for patients consistently without ILAs (Fig. 3. **a**) and those with new ILAs (Fig. 3. **b**)

**Fig. 4:**
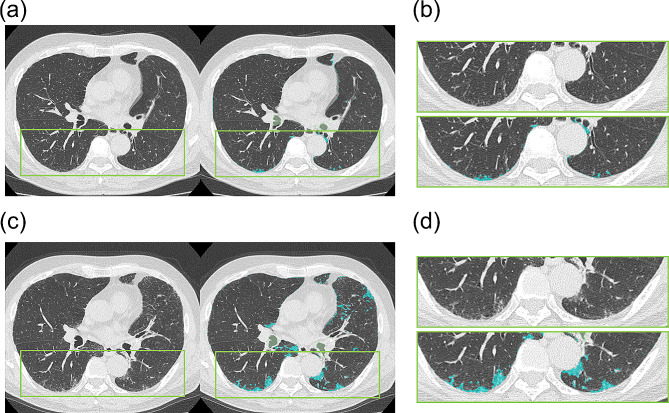
Representative images of a patient without ILA at enrollment who had ILA at follow-up Legend: Representative images are of a 77-year-old male patient who developed ILAs according to the follow-up CT scan. The patient did not have ILAs at baseline (shown in Fig. 4. **a** and Fig. 4. **b**), but some emerged at follow-up (shown in Fig. 4. **c** and Fig. 4. **d**). In the right panels of Fig. 4. **a** and Fig. 4. **c** and the lower panels of Fig. 4. **b** and Fig. 4. **d**, ILD regions segmented as GGOs, reticulations, and honeycombing using AIQCT are overlaid onto the CT images in light blue. The ILD volume was 47.3 ml (0.98%) at baseline and increased to 164 ml (3.4%) at follow-up

124 patients were included in the longitudinal analysis who took follow-up CT scans in 5 ± 1 years after the baseline scans (Fig. 1). Eight (7%) patients demonstrated new ILAs on the follow-up CT (mean CT scan interval = 4.9 ± 0.5 years) (Fig. S2). When comparing the baseline characteristics of patients consistently without ILAs and those with new ILAs (Table 3), pulmonary function and AIQCT indices were not different between the groups. Figure 3 shows the longitudinal changes in the AIQCT indices. The ILDvol and the volumes of its components (honeycombing, reticulations, and GGOs) increased in patients with new ILAs at the follow-up CT scan. The volumes of nodules also increased in patients with new ILAs at the follow-up, which may represent an increase in the number of nodules. The volume changes in these indices were greater in patients with new ILAs than in those consistently without ILAs after adjusting for age, sex, BMI, and smoking exposure (Table 4). Bronchial volume tended to increase in patients with new ILAs, although the difference did not reach statistical significance (Table 4). Representative images illustrating the ILAs monitored during the follow-up period are shown in Fig. 4.

**Table 3:** Baseline characteristics of patients with COPD without ILAs at enrollment who subsequently developed ILAs and those who consistently showed no ILA findings on follow-up CT scans

	Consistently without ILA		new ILA		P value
n	103		8		
Follow-up duration, y	4.9	± 0.5	5.2	± 0.3	0.07
Age, y	69.1	± 7.6	74.98	± 8.2	0.04
Sex, male	94	(91.3)	8	(100)	0.842
BMI, kg/m^2^	21.8	± 2.7	23.8	± 1.5	0.035
Pack-years	64.5	± 34.9	50.4	± 23.8	0.266
Smoking status, past	74	(71.8)	6	(75)	1
mMRC, ≥2	27	(26.2)	2	(25)	1
FVC, L	3.23	± 0.77	3.09	± 0.69	0.601
% predicted	93.6	± 17.7	97.1	± 22.7	0.59
FEV_1_, L	1.57	± 0.62	1.56	± 0.52	0.981
% predicted	58.3	± 19.9	63.3	± 18.1	0.49
FEV_1_/FVC, %	47.9	± 12.9	50.2	± 9.1	0.625
D_LCO_, mL/min/mmHg	13.35	± 5.02	12.24	± 3.16	0.541
% predicted	60.5	± 20. 7	61.2	± 18.8	0.929
Normal lungs, mL	4226	± 897	3966	± 929	0.432
ILD volume, mL	52.4	± 38.6	49.4	± 13.7	0.828
Ground-glass opacities, mL	45.1	± 35.7	38.4	± 9.7	0.596
Reticulations, mL	6.3	± 6.2	10.2	± 5.2	0.084
Consolidations, mL	7.6	± 3.7	8.1	± 2.3	0.704
Honeycombing, mL	1.0	± 1.7	0.86	± 1.3	0.802
Nodules, mL	5.7	± 4.6	6.6	± 4.4	0.575
Hyperlucencies, mL	808	± 994	599	± 674	0.561
Bronchi, mL	76.8	± 20.8	74.2	± 17.2	0.739
Vessels, mL	212.2	± 55.2	199.8	± 65.6	0.547

Data are the mean ± standard deviation or the number of patients with the percentage in parentheses. *ILA* interstitial lung abnormality; *BMI* body mass index; *mMRC* modified British Medical Research Council Dyspnea scale; *FVC* forced vital capacity; *FEV*_1_, forced expiratory volume in 1 s; *DLCO* diffusing capacity of the lung for carbon monoxide; *ILD* interstitial lung disease

**Table 4:** Longitudinal changes in AIQCT indices

	Consistently without ILAs	New ILAs	Adjusted difference	P value	
n	103	8			
ILD volume, mL	-2.51 ± 33.45	204.20 ± 235.75	197.2 [145, 249.4]	< 0.001	
%	-0.11 ± 0.96	4.64 ± 5.46	4.6 [3.3, 5.9]	< 0.001	
Ground-glass opacity, mL	-5.31 ± 29.90	110.11 ± 163.87	111.9 [72.6, 151.2]	< 0.001	
%	-0.17 ± 0.90	2.63 ± 4.30	2.8 [1.7, 3.8]	< 0.001	
Reticulation, mL	1.91 ± 6.72	85.98 ± 160.76	78.6 [46.6, 110.5]	< 0.001	
%	0.04 ± 0.15	1.83 ± 3.39	1.7 [1, 2.4]	< 0.001	
Honeycombing, mL	0.89 ± 4.19	8.11 ± 12.77	6.7 [2.7, 10.7]	< 0.001	
%	0.02 ± 0.08	0.18 ± 0.27	0.1 [0.1, 0.2]	< 0.001	
Consolidation, mL	4.75 ± 30.81	5.17 ± 8.59	-0.2 [-23.4, 23]	0.99	
%	0.08 ± 0.53	0.11 ± 0.18	0 [-0.4, 0.4]	0.90	
Nodules, mL	0.29 ± 4.39	8.29 ± 13.62	8.2 [4.2, 12.3]	< 0.001	
%	0.00 ± 0.12	0.17 ± 0.30	0.2 [0.1, 0.3]	< 0.001	
Bronchi, mL	3.15 ± 14.79	15.00 ± 30.70	10.8 [-1.8, 23.3]	0.09	
%	0.07 ± 0.32	0.32 ± 0.69	0.2 [-0.1, 0.5]	0.13	
Vessels, mL	-7.26 ± 59.18	14.32 ± 87.75	27.1 [-19.8, 74]	0.25	
%	-0.12 ± 1.17	0.38 ± 1.94	0.7 [-0.3, 1.6]	0.17	
Hyperlucency, mL	253.80 ± 467.99	236.01 ± 394.52	162.1 [-167.6, 491.8]	0.33	
%	4.95 ± 10.50	4.76 ± 7.89	3.8 [-3.5, 11.1]	0.30	

Data are the mean ± standard deviation or adjusted mean differences with 95% confidence intervals in parentheses. The differences between patients who did not develop ILA and those who developed ILA were adjusted by age, sex, BMI, smoking status, and cumulative smoking exposure. *ILA* interstitial lung abnormality; *ILD* interstitial lung disease

When using the optimal ILDvol% threshold defined in the cross-sectional analysis (1.203%), 6 out of 8 patients were classified as having no ILAs at baseline, but all were classified as having ILAs at follow-up.

## Discussion

This study showed that AI-based quantitative analysis identified COPD with ILAs with high sensitivity and specificity. Furthermore, ILAs appeared in 7% of patients with COPD 5 years after baseline CT acquisition. The volumes of not only GGOs, reticulations, and honeycombing but also nodules increased in patients with new ILAs. This is the first study to classify the findings from chest CT scans into detailed lesions, including airway lesions and to quantitatively analyze and longitudinally assess patients with COPD and ILAs.

The prevalence of ILAs in patients with COPD in this study was 10 to 14%, which was consistent with the previous reports [20, 21]. ILAs in patients with COPD are more symptomatic, frequently exacerbated, and have a poor prognosis [8, 20, 22]. Therefore, the effort to detect ILAs in patients with COPD is crucial for understanding and managing disease progression. Quantitative interstitial features assessed by local histogram- and distance-based method are associated with pulmonary functions, respiratory symptoms, and mortality in patients with COPD [23]. The increase of quantitative interstitial features is associated with an annual decline in FVC, a decrease in exercise tolerance, and a poor mortality in longitudinal assessments [11]. Furthermore, the progression of interstitial changes quantified using deep learning-based method had further impact on mortality in addition to the progression of emphysema in the COPD cohort [24]. This study extends those findings by providing a longitudinal, quantitative assessment of patients with new ILAs via a detailed classification of chest CT scans using AIQCT.

AIQCT achieved good diagnostic performance with high sensitivity and specificity in identifying ILAs. This result is comparable to that from previous reports, although the quantifying techniques differed [25–27]. The threshold values in this study (1.203%) and previous reports (1.8 to 3.6% [25–27] are also lower than the threshold value for visual assessment proposed by the Fleischner Society (5%) [7]. The visual quantification of ILD volume based on tomographic images might be difficult, and the volume itself is usually overestimated [27]. In the sensitivity analysis, the diagnostic performance was not improved even when the lung volume was divided. This might be because ILD lesions are usually localized in the basal part of the lung in patients with ILAs, and artifacts on CT images did not affect the diagnosis of quantitative ILAs.

ILAs emerged in 7% of patients with COPD within 5 years in this study. The prevalence of new ILAs was higher than that in a population-based study [28]. This study included more at-risk patients, that is, older patients and those with greater smoke exposure [29]. The baseline pulmonary function and volumes of CT regions did not differ between patients who developed ILAs and those who consistently remained without ILAs, which indicates that the occurrence of ILAs cannot be predicted by baseline pulmonary function or AIQCT indices.

The longitudinal changes in volumes of ILD regions and nodules were greater and that in bronchial volumes tended to be greater in patients with new ILAs than in those consistently without ILAs. Recently, some studies have shown that small airways are important to the pathogenesis of ILD or ILAs [30–33]. The microCT analysis of unbiased, systematic uniform random tissue samples from patients with IPF showed that the terminal bronchiole was dilated even in the presence of lesions without fibrosis [31]. The airway wall on HRCT is thicker in subjects with ILAs than in those without ILAs [30]. Furthermore, bronchiectasis and peribronchial fibrosis were identified in ILA lungs in histopathological studies [32]. These airway changes may appear as nodules due to the resolution of HRCT. This study supports the hypothesis that interstitial fibrotic changes may start in the bronchial regions.

The interrater agreement was not high, neither between CT-experienced pulmonologists or with respect to a chest radiologist. Airspace enlargement with fibrosis, scar-like lesions, and paraseptal emphysema may make it harder to assess the existence of ILAs, especially in patients with COPD [34]. AIQCT is useful for providing objective and reproducible assessments of even slight interstitial abnormalities in emphysematous patients. Furthermore, it can detect the progression of ILAs. To elucidate the pathogenesis of ILAs and their clinical sequences to ILD, repeated histological analyses are needed. However, biopsy is too invasive to repeat over time, and the biopsy procedure may alter the microenvironment of the lung. In contrast, CT image analysis is less invasive and allows repeated examinations without changing the lung microenvironment. Imaging studies using AIQCT may elucidate the pathogenesis of ILAs.

This study has some limitations. First, the number of new ILAs was small, making it is difficult to explore factors associated with the future development of ILAs. Second, a substantial number of patients did not undergo follow-up CT scans within the period. A selection bias may exist when patients undergo follow-up CT scans. Third, most patients included in this study were male because COPD patients tend to be male in Japan and Asian countries [35, 36].

## Conclusions

In conclusion, this study showed that ILAs in patients with COPD could be measured using AIQCT. 7% of patients with COPD showed new ILAs at follow-up, and these patients could be identified by using AIQCT. Although additional studies in longitudinal cohorts are needed, AIQCT revealed that honeycombing, reticulation, GGO, and nodule volumes were greater in patients who developed ILAs.

### Electronic supplementary material

Below is the link to the electronic supplementary material.

Supplementary Material 1

## Data Availability

All data generated or analyzed during this study are included in this article and its supplementary material files. Further enquiries can be directed to the corresponding author.

## Abbreviations

ILA: interstitial lung abnormality
COPD: chronic obstructive pulmonary disease
AI: artificial intelligence
ILD: interstitial lung disease
CPFE: combined pulmonary fibrosis and emphysema
HRCT: high-resolution computed tomography
AIQCT: Artificial intelligence based quantitative CT image analysis software
GGO: ground-glass opacity
IPF: idiopathic pulmonary fibrosis
FEV_1_: forced expiratory volume in 1 s
FVC: forced vital capacity
