# Supplementary material for: Longitudinal assessment of interstitial lung abnormalities on CT in patients with COPD using artificial intelligence-based segmentation: a prospective observational study
Source: BMC Pulm Med. 2024 Apr 23;24:200. doi: 10.1186/s12890-024-03002-z (PMC11036664; doi:10.1186/s12890-024-03002-z)
Supplement: Supplementary file 1 — Supplementary Material 1 [file 12890_2024_3002_MOESM1_ESM.docx]

**Additional files**

**Supplementary Methods**

**Validation cohort**

To validate the threshold of ILDvol% for diagnosing ILAs, the data from another cohort were used. This validation cohort was a prospective observational cohort from Kyoto university hospital and a respiratory clinic (Terada Clinic), consisting of smokers aged ≥40 years with a history of ≥10 pack-years between April 2018 and April 2020 [1]. The exclusion criteria were (1) a history of other respiratory diseases, such as interstitial lung disease and lung cancer, (2) a current primary diagnosis of asthma, (3) α1-antitrypsin deficiency, and (4) lung surgical resection. Patients who were enrolled in both the study cohort and the validation cohort were excluded from the validation cohort in this study. Patients in the validation cohort who did not meet the GOLD criteria were also excluded from this study [2]. Full inspiration chest CT scans were obtained in this study, although end-tidal expiration chest CT scans were also acquired in this validation cohort. An Aquilion Presicion scanner (Canon Medical Systems, Otawara, Japan) was used at the hospital, and an Aquilion lightning scanner (Canon Medical Systems, Otawara, Japan) was used at the clinic. The CT conditions of both scanners were as follows: 120 kVp, 0.5-s exposure time, and autoexposure control. Images with a 1 mm slice thickness are reconstructed using a sharp kernel.

**Sensitivity analysis**

Sensitivity analysis was conducted to assess whether the diagnostic performance could be improved when ILDvol% was calculated in equally divided lung volumes into one-third or 10%. Total lung volume was calculated using chest CT scans. The volume of each slice was summed along with the craniocaudal axis until it reached the nearest one-third or 10% of the total lung volume. ROC curve analysis used the highest ILDvol% in each fraction of lung volumes.

**References**

1. Tanabe N, Shimizu K, Terada K, Sato S, Suzuki M, Shima H, et al. Central airway and peripheral lung structures in airway disease-dominant COPD. ERJ Open Res. 2021 Jan;7(1):00672–2020.
2. GLOBAL INITIATIVE FOR CHRONIC OBSTRUCTIVE LUNG DISEASE GLOBAL STRATEGY FOR THE DIAGNOSIS, MANAGEMENT, AND PREVENTION OF CHRONIC OBSTRUCTIVE PULMONARY DISEASE (2023 REPORT). 2022.Available from: [www.goldcopd.org](http://www.goldcopd.org)

**Table S1. Clinical and radiological comparisons between patients with and without ILAs in the validation cohort**

|  | No ILAs |  | ILAs |  | *P* value |
| --- | --- | --- | --- | --- | --- |
| n | 138 |  | 15 |  |  |
| Age, y | 71.3 | ± 8.3 | 77.2 | ± 7.5 | 0.009 |
| Sex, male | 129 | (93.5) | 13 | (86.7) | 0.657 |
| BMI, kg/m^2^ | 22.9 | ± 3.7 | 24.7 | ± 5.1 | 0.085 |
| Pack-years | 59.3 | ± 30.5 | 55.3 | ± 25.5 | 0.626 |
| Smoking status, past | 99 | (71.7) | 12 | (80.0) | 0.707 |
| mMRC, ≥2 | 33 | (23.9) | 4 | (26.7) | 1.0 |
| FVC, L | 3.02 | ± 0.91 | 3.18 | ± 1.03 | 0.527 |
| % predicted | 86.1 | ± 21.8 | 98.5 | ± 20.1 | 0.037 |
| FEV_1_, L | 1.63 | ± 0.68 | 1.92 | ± 0.63 | 0.12 |
| % predicted | 60.7 | ± 23.0 | 78.1 | ± 17.5 | 0.005 |
| FEV_1_/FVC, % | 53.0 | ± 12.0 | 61.0 | ± 8.0 | 0.019 |
| Normal lungs, mL | 4557 | ± 1170 | 3830 | ± 1134 | 0.023 |
| ILD volume, mL | 53.6 | ± 45.5 | 110.6 | ± 44.2 | <0.001 |
| Ground-glass opacities, mL | 47.0 | ± 39.8 | 71.7 | ± 30.5 | 0.02 |
| Reticulations, mL | 6.0 | ± 8.3 | 28.6 | ± 15.7 | <0.001 |
| Consolidations, mL | 8.8 | ± 6.8 | 10.6 | ± 4.8 | 0.32 |
| Honeycombing, mL | 0.6 | ± 1.1 | 10.4 | ± 26.1 | <0.001 |
| Nodules, mL | 4.6 | ± 3.8 | 7.8 | ± 5.1 | 0.003 |
| Hyperlucencies, mL | 898.2 | ± 1035 | 760.9 | ± 941 | 0.624 |
| Bronchi, mL | 82.1 | ± 20.3 | 99.3 | ± 25.2 | 0.003 |
| Vessels, mL | 233.5 | ± 65.3 | 226.4 | ± 86.3 | 0.698 |

Data are the mean ± standard deviation or the number of patients with percentage in parentheses.

ILA, interstitial lung abnormality; BMI, body mass index; mMRC, modified British Medical Research Council Dyspnea scale; FVC, forced vital capacity; FEV_1_, forced expiratory volume in 1 second; DLCO, diffusing capacity of the lung for carbon monoxide; ILD, interstitial lung disease

**Figure S1. The optimal threshold of ILD volume for predicting ILA as determined by using receiver operating characteristic curve analysis in the whole lung and after dividing the lung into three or ten zones.**


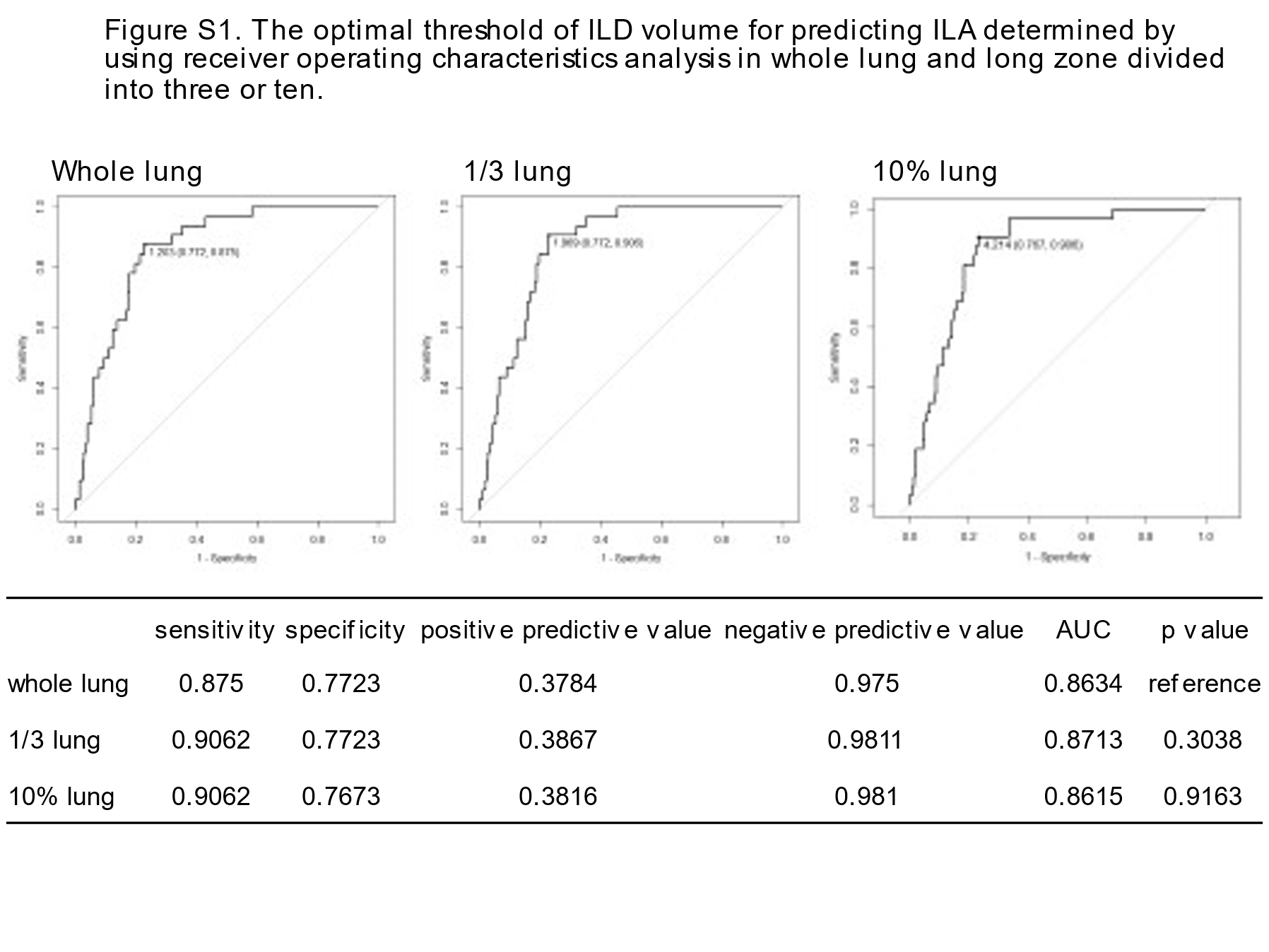


The optimal ILD volume threshold in the whole lung was 1.203%, with an area under the ROC curve (AUC) of 0.863, 87.5% sensitivity, and 77.2% specificity. When the lung volume was equally divided into three or ten parts along the craniocaudal axis, the optimal ILD volume thresholds were 1.969% and 4.214%, respectively, both demonstrating high sensitivity and specificity. However, the AUCs were not different when comparing the results using the DeLong test.

**Figure S2. Longitudinal changes in the visual assessment of ILA.**


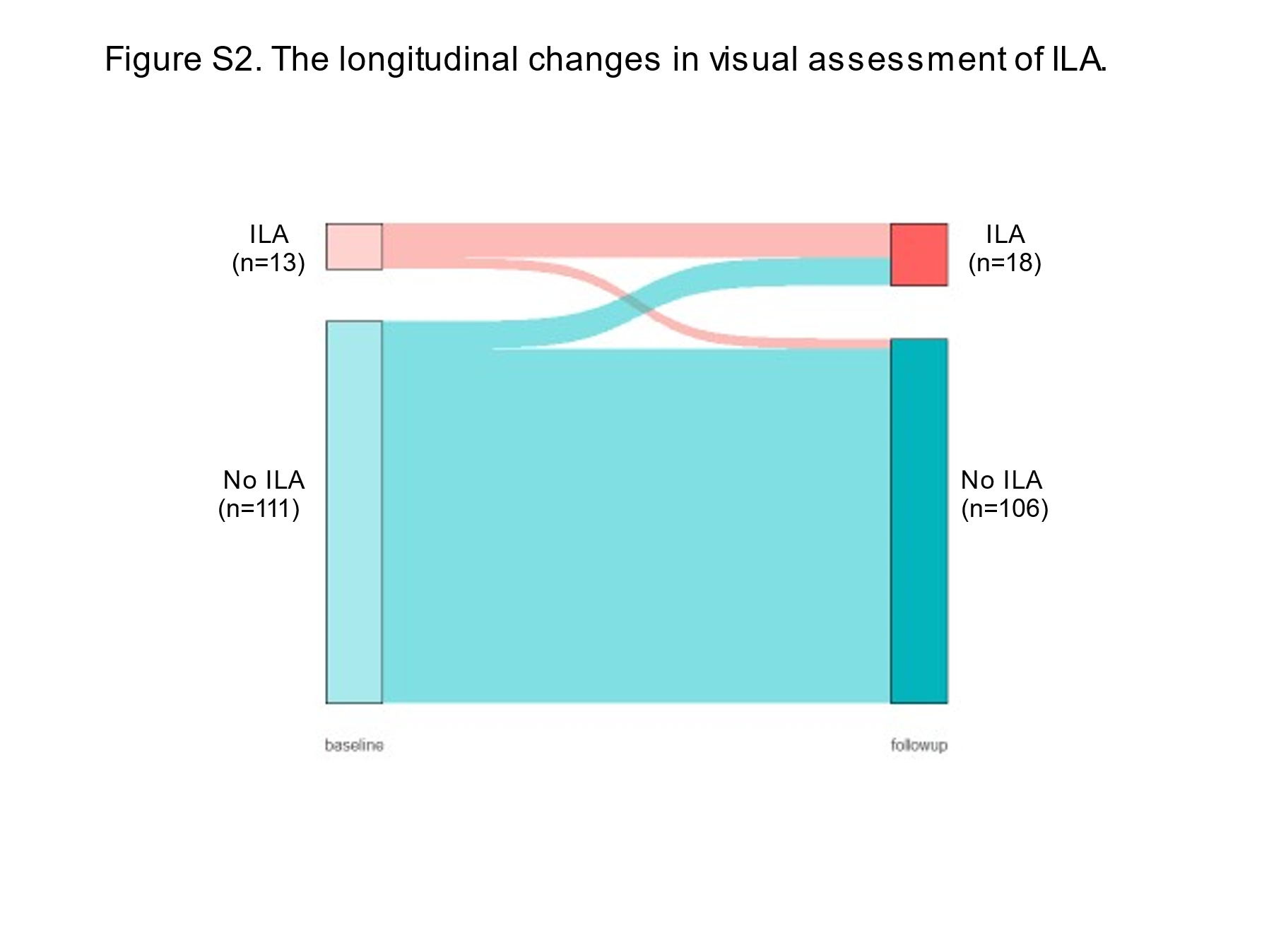


Among 124 patients, 13 patients had ILAs, and 111 patients did not have ILAs at baseline, while 18 patients had ILAs, and 106 patients did not have ILAs on follow-up CT. ILAs emerged in 8 patients at follow-up. Three out of 13 patients with ILAs at baseline did not have ILA on follow-up CT, while 10 patients consistently had ILAs.
